# Intermittent ventricular pacing suppression owing to P-wave oversensing with automatic sensitivity adjustment

**DOI:** 10.1016/j.hrcr.2026.07.016

**Published:** 2026-07-30

**Authors:** Takafumi Sasaki, Masao Takahashi, Koichiro Yamaoka, Tomoyuki Arai, Rintaro Hojo, Seiji Fukamizu

**Affiliations:** Department of Cardiology, Tokyo Metropolitan Hiroo Hospital, Tokyo, Japan

**Keywords:** P-wave oversensing, Automatic sensitivity adjustment, Generator replacement, VDD pacemaker, Far-field sensing, Ventricular pacing inhibition, Pacemaker programming, Tricuspid annulus

**Key Teaching Points**

• New-onset ventricular pacing loss after generator replacement should raise suspicion of oversensing caused by a newly introduced automatic sensitivity algorithm, not only lead dislodgement.
• A long bipolar electrode spacing and a ring electrode close to the tricuspid annulus predispose to far-field P-wave oversensing. The same mechanism occurs with integrated bipolar implantable cardioverter-defibrillator leads and coronary sinus leads.
• Intracardiac electrogram review is essential to confirm that the oversensed signal is locked to atrial activity and to exclude lead and noise-related causes.
• Reprogramming to unipolar (tip–can) sensing, which excludes the ring electrode, can eliminate P-wave oversensing when raising the sensitivity value alone fails.
• Patient-specific programming is preferable to default “out-of-the-box” settings, especially when an automatic sensitivity algorithm is newly introduced.

## Introduction

Cardiac device sensing has 2 types: fixed sensitivity and automatic sensitivity. P-wave oversensing (PWOS) is very rare because of the widespread use of fixed-sensitivity pacemakers and post-atrial ventricular blanking periods to prevent crosstalk.^1^^,^^2^ Azure XT (Medtronic, Minneapolis, MN) employs automatic sensitivity adjustment, and we have previously reported T wave oversensing with Azure XT.^3^ We now report a clinically significant case of PWOS with Azure XT, which, to our knowledge, has not been previously described in this device.

## Case report

An 87-year-old woman, diagnosed with a complete atrioventricular block in 1995, presented with a Medtronic VDD pacemaker with a right ventricular lead (CapSure VDD, model 5032; Medtronic) implanted at another hospital and an atrial lead added in 2011 due to atrial lead failure. She was admitted in September 2020 for generator replacement because of battery depletion. On admission, she was 145 cm tall and weighed 42 kg. Transthoracic echocardiography showed normal ejection fraction and no significant valvular disease. Chest radiographs showed no significant findings associated with the device.

Generator replacement was performed on the second day. The implanted generator was a Azure XT DR MRI SureScan (W2DR01; Medtronic; Minneapolis, MN), programmed in DDD mode with a lower rate of 60 pulses per minute, AV delay (sensed and paced) of 150 and 180 ms, post-ventricular atrial blanking (PVAB) of 150 ms, and ventricular sensitivity set at the nominal value of 0.9 mV with automatic sensitivity adjustment enabled. The procedure was completed without any adverse events, and no significant findings were observed on pre- and postoperative radiographs. No changes in lead parameters (pacing threshold, sensing amplitude, or impedance) before and after the procedure were observed. However, 2 h after the procedure, intermittent loss of ventricular pacing appeared on the monitor (Figure 1A). When we checked the intracardiac electrogram (EGM), we recorded a far-field P wave at the tip-ring bipolar electrode of the RV lead, and intermittent oversensing of that far-field P wave—temporally locked to intrinsic atrial activity on the EGM—suppressed ventricular pacing (Figure 1B). There were no findings suggestive of lead dislodgement, electromagnetic interference, myopotential oversensing, or lead conductor failure (lead impedance remained stable and no short-interval noise counts were observed). We changed the ventricular sensitivity from 0.9 mV to 2 mV, but PWOS persisted. Checking the unipolar electrodes of the ventricular lead revealed that far-field P wave potentials were recorded only at the ring electrode. Ventricular sensing was then programmed to unipolar (tip–can) configuration, which successfully eliminated PWOS. The patient was discharged from the hospital 5 days after surgery without any complications, and no recurrence of PWOS was observed at the outpatient follow-up visit.

**Figure 1:**
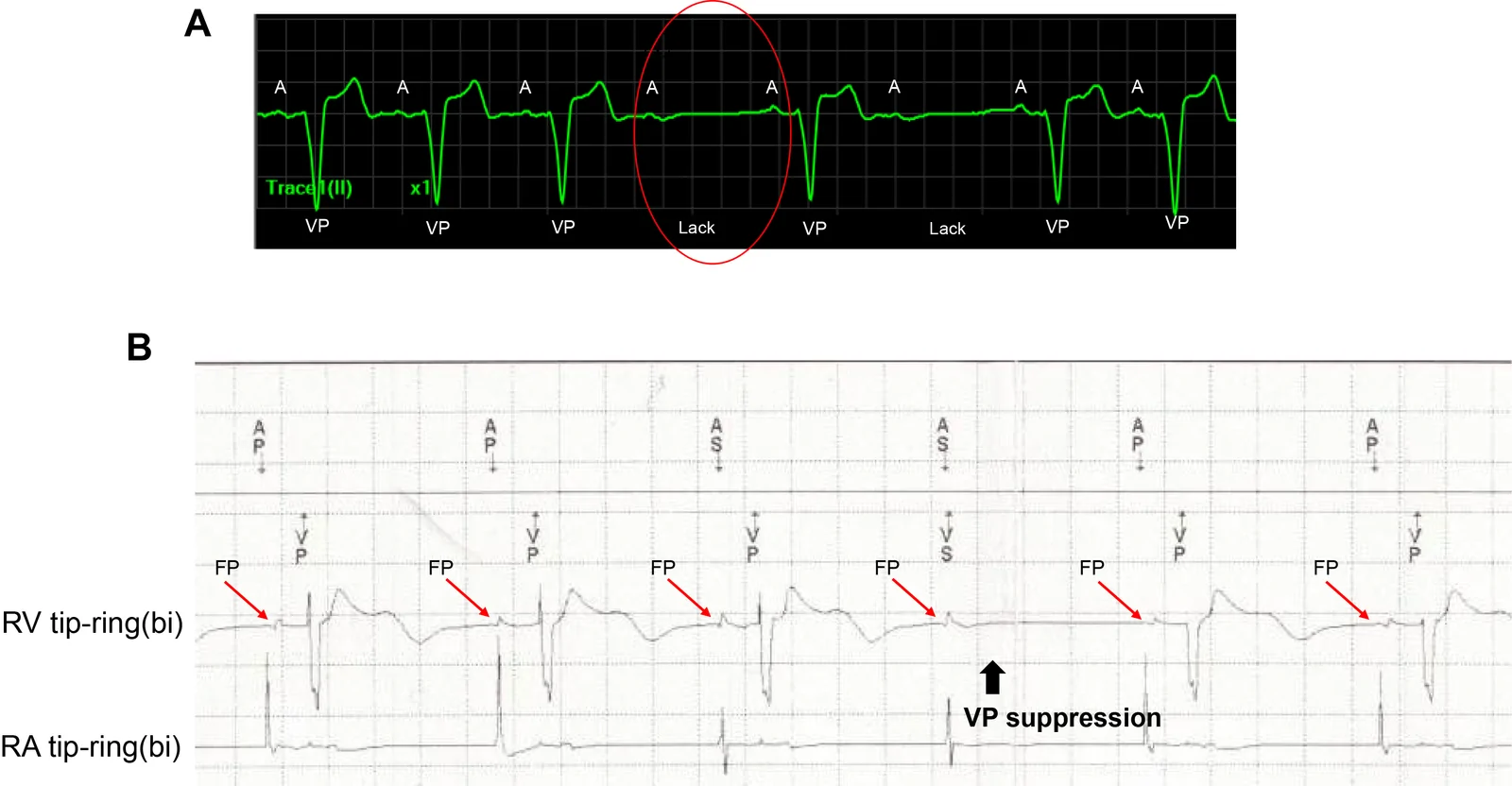
**A:** Postoperative electrocardiogram monitor (lead Ⅱ): lack of ventricular pacing. **B:** Intracardiac electrogram of right atrium and right ventricular leads and bipolar tip-ring: suppressed ventricular pacing because of intermittent oversensing of far-field P wave. FP = far-field P wave; RA = right atrium; RV = right ventricle; VP = ventricular pacing.

## Discussion

We believe that the 2 important contributing factors to PWOS in this case were the change from fixed to automatic sensitivity adjustment and the long bipolar electrode spacing of the VDD lead. The cumulative ventricular pacing rate was 99.9%, and the previous generator with fixed sensitivity was replaced with a new generator with automatic sensitivity adjustment. The automatic sensitivity adjustment algorithm of Azure XT may have facilitated oversensing under these specific lead and anatomical conditions, as explained below. The automatic sensitivity adjustment of Azure XT is different after ventricular sensing and after pacing. After ventricular sensing, when the nominal set sensitivity is 0.9 mV and the time constant is 450 ms, the sensitivity is gradually reduced from 9 mV, 10 times the set sensitivity, to the set sensitivity according to the time constant. The sensitivity will not fall below the set sensitivity. Conversely, after ventricular pacing, when the nominal sensitivity is 0.9 mV and the time constant is 450 ms, after 200 ms of blanking from the V pace, the sensing threshold decays from 1.8 mV to 0.9 mV (increasing sensitivity) of the set sensitivity according to the time constant. The postoperative heart rate was 60–80 beats per minute, and the time from ventricular pacing to the far-field P wave was 550–800 ms, which would have resulted in a sensitivity of approximately 0.9 mV. The far-field P wave height was 0.6–1.0 mV, and PWOS was considered to have occurred when the far-field P wave exceeded the sensitivity curve (Figure 2).^3^ The CapSure VDD lead used in this case has a tip–ring spacing of 30 mm, which is longer than that of conventional ventricular leads. Although the relationship between tip-to-ring spacing and far-field signal amplitude has been established primarily for atrial leads,^4^ the same principle is likely applicable to ventricular leads with long bipolar spacing, potentially increasing susceptibility to far-field atrial signal detection. Cine imaging and echocardiography showed that the ring electrode was located near the tricuspid annulus (Figure 3A and 3B). The far-field P wave was observed only in the sensing polarity where the ring electrode was included, further supporting the conclusion that the ring electrode’s anatomical proximity to the tricuspid annulus was a key predisposing factor. Barold et al previously reported PWOS caused by displacement of a bipolar ventricular lead toward the right ventricular inflow tract^2^; in our case, the anatomical proximity of the ring electrode to the tricuspid annulus appears to be the analogous predisposing factor. The same phenomenon of far-field atrial signal oversensing by the ventricular sensing channel has been described in other lead configurations in which a sensing electrode lies in close proximity to atrial tissue. In implantable cardioverter-defibrillators with integrated bipolar leads, P-wave oversensing can occur when the distal shocking coil straddles or lies near the tricuspid valve and the sensed PR interval exceeds the ventricular blanking period,^5^ and in cardiac resynchronization therapy devices, far-field atrial oversensing by a left ventricular lead positioned in the coronary sinus can inhibit biventricular pacing.^6^ These reports share the common mechanism of a widely spaced sensing vector capturing far-field atrial depolarization, consistent with the present case. In this case, PWOS was eliminated by programming ventricular sensing to a unipolar (tip–can) configuration, which excluded the ring electrode from the sensing vector (Figure 3C). Although PWOS in pacemakers is very rare,^1^^,^^2^^,^^7^ it can occur in pacemakers with long bipolar ventricular leads and automatic sensitivity adjustment, as in this case. To our knowledge, PWOS specifically attributable to automatic sensitivity adjustment has been reported in only limited contexts. Van Puyvelde et al recently described far-field PWOS during left bundle branch area pacing using Biotronik’s Automatic Sensitivity Control algorithm,^8^ and a near-fatal PWOS was reported in a Boston Scientific Ingenio device operating in Safety Mode with automatic gain control.^9^ However, PWOS caused by the Medtronic Azure XT automatic sensitivity adjustment in combination with a long bipolar VDD lead has not been previously reported, highlighting the unique mechanism in the present case. Furthermore, experts in conduction system pacing have recommended using fixed sensitivity rather than automatic sensitivity for His bundle pacing, precisely because automatic sensitivity can lower the sensing threshold to levels prone to oversensing of low-amplitude signals.^10^ This principle is equally applicable to the present scenario. Clinicians should be aware that generator replacement with a device employing automatic sensitivity adjustment, particularly in patients with long bipolar leads positioned near the tricuspid annulus, carries a risk of PWOS. In such cases, careful post-procedural monitoring and prompt evaluation of intracardiac electrograms are essential. Importantly, this complication may be avoidable by careful inspection of the intracardiac electrograms at the time of implantation or generator replacement, rather than relying on default “out-of-the-box” programming. Patient-specific programming—tailored to the individual lead type, electrode spacing, and anatomical position—is essential to prevent oversensing, particularly when an automatic sensitivity algorithm is newly introduced.

**Figure 2:**
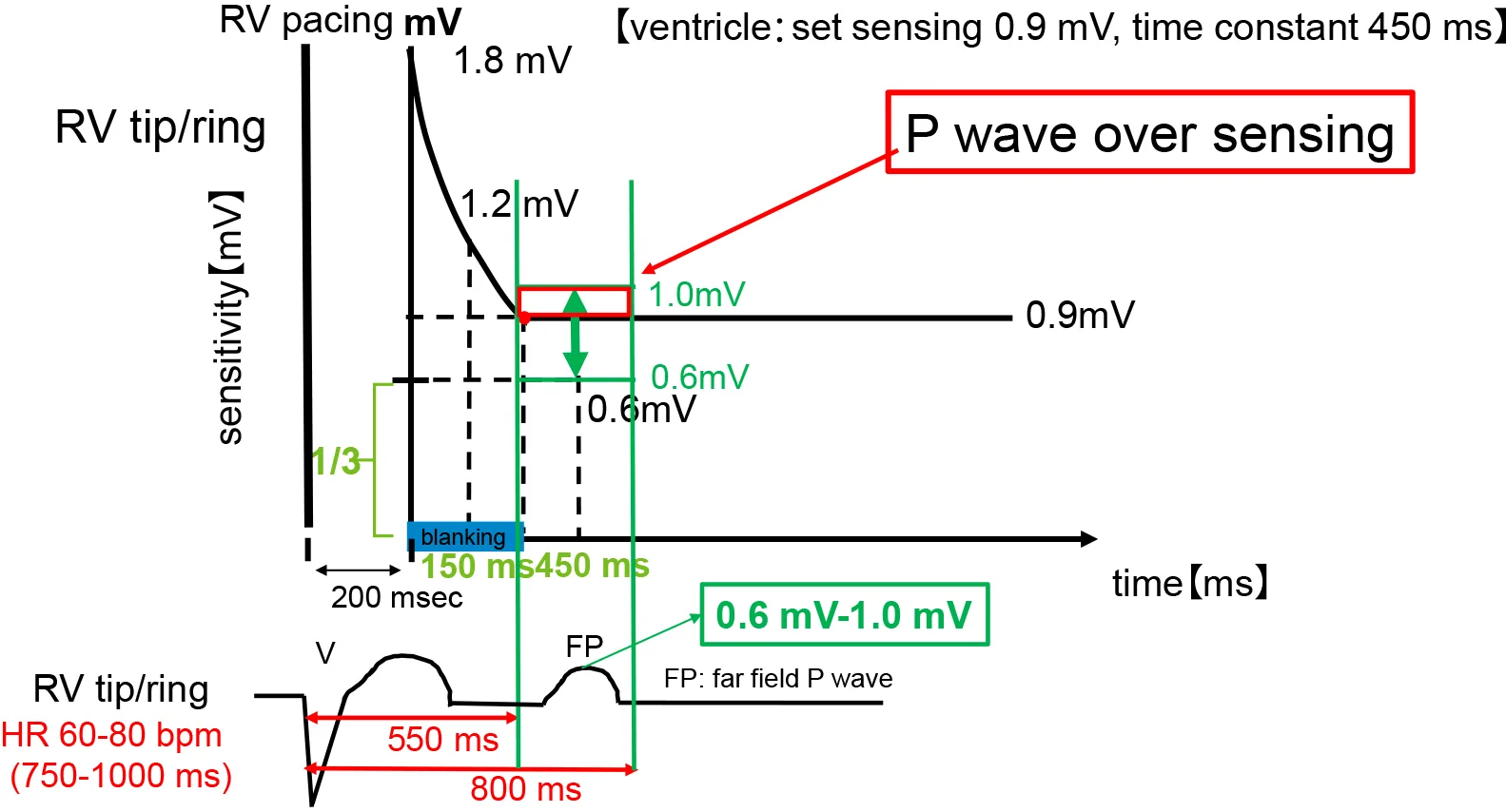
Auto-adjustment sensitivities of the pacemakers used during ventricular pacing: Sensing is blanked for the first 200 ms after ventricular pacing and then increases over time with a time constant of 450 ms, never falling below the nominal sensitivity. In this case, the ventricular-paced event (VP) rate was 99.9%; therefore, automatic sensitivity adjustment at V pace was used. Since the pulse rate is approximately 60–80 beats per minute, the time between VP and far-field potential in this case is approximately 550–800 ms, and the sensitivity at that time would have been approximately 0.9 mV. The far-field P wave height ranged from 0.6 mV to 1.0 mV, and P-wave oversensing was considered to have occurred when the far-field P wave exceeded the sensitivity curve. Adapted from Takahashi et al,^3^ with permission from John Wiley and Sons. FP = far-field P wave; RV = right ventricle.

**Figure 3:**
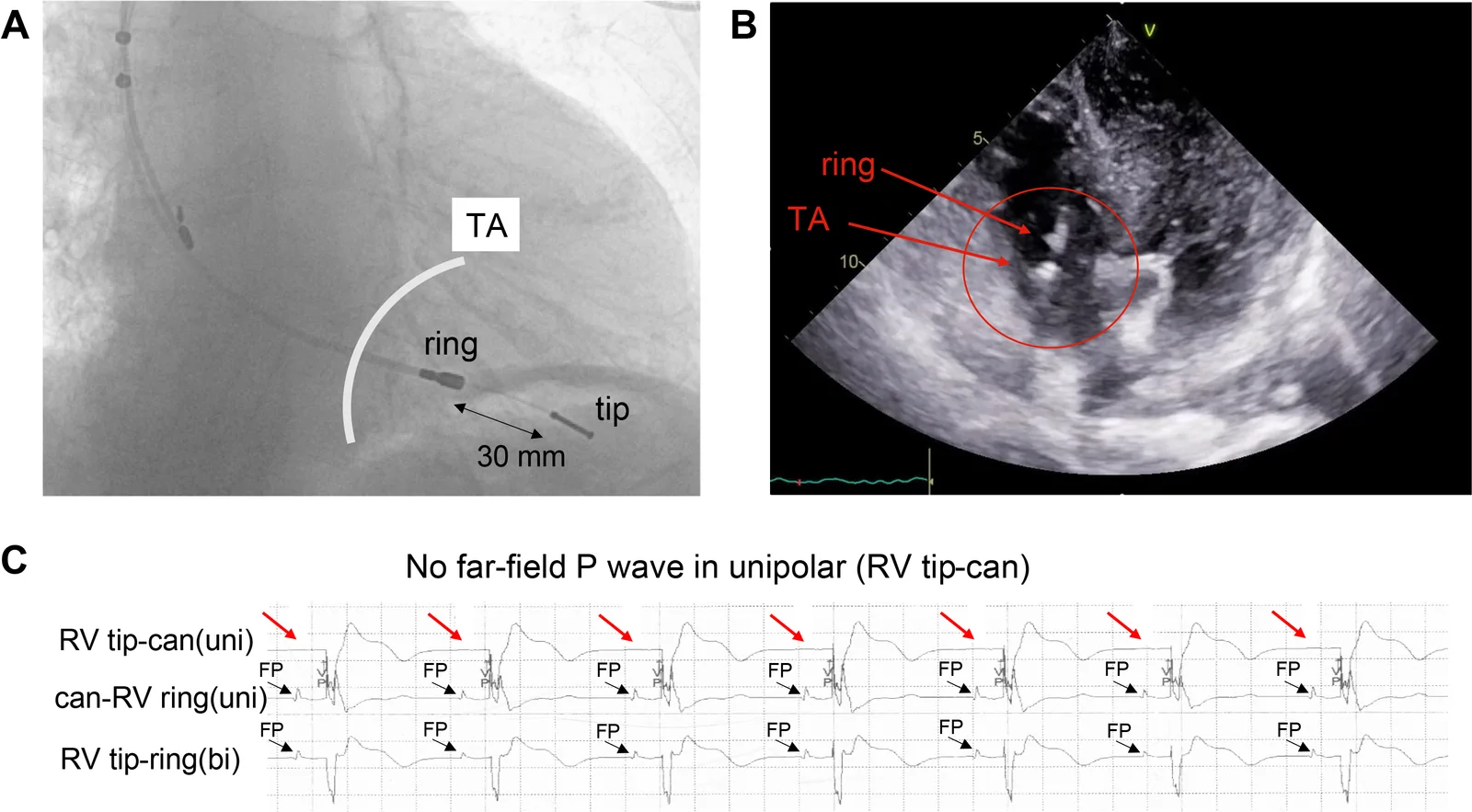
Why was the far-field P wave observed in the V lead? The CapSure VDD used in the present case was located in the vicinity of the tricuspid valve annulus (TA), with a distance of 30 mm between the tip and ring. **A:** Cine image of the position of the TA with the tip and ring. **B:** Transthoracic echocardiogram of the location of the TA and ring electrode. **C:** Intracardiac electrogram: Can-ring and tip-ring showed far-field P wave (FFPW); however, tip-can without ring did not exhibit FFPW, indicating that the ring electrode was located in the area near the TA. AP = atrial pacing; AS = atrial sensing; bi = bipolar; FP = far-field P wave; RA = right atrium; RV = right ventricle; uni = unipolar; VP = ventricular pacing.

## Conclusion

PWOS may occur when a long bipolar lead is used with a pacemaker that has automatic sensitivity adjustment, such as the Azure XT, particularly when the ring electrode is anatomically close to the tricuspid annulus. When a new onset of ventricular pacing loss is observed after generator replacement, clinicians should consider not only lead dislodgement but also oversensing due to newly introduced sensing algorithms. Intracardiac EGM review and programming of ventricular sensing to unipolar (tip–can) configuration, which excludes the ring electrode, should be considered as an early diagnostic and therapeutic step.
